# Impact of antimicrobial use in dogs on antimicrobial resistance and shared flora with human owners

**DOI:** 10.1017/ash.2022.323

**Published:** 2023-01-09

**Authors:** Kirthana Beaulac, Claire L. Fellman, Annie S. Wayne, Laura A. McDermott, David R. Snydman, Shira Doron

**Affiliations:** 1 Tufts Medical Center, Boston, Massachusetts; 2 Department of Clinical Sciences, Cummings School of Veterinary Medicine, Tufts University, North Grafton, Massachusetts; 3 Division of Infectious Diseases and Geographic Medicine, Tufts Medical Center, Boston, Massachusetts (Present affiliations: Department of Pharmacy, Emerson Hospital, Concord, Massachusetts, [K.B.] and Massachusetts Veterinary Referral Hospital, Woburn, Massachusetts [A.S.W.])

**Keywords:** antimicrobial resistance, antimicrobial stewardship, One Health

## Abstract

Transmission of bacteria between animals and humans in domestic households is increasingly recognized. We evaluated the presence of antimicrobial-resistant fecal bacteria in 8 dog-owner–dog pairs before and after the dog received amoxicillin-clavulanate. The study identified shared flora in the humans and dogs that were affected by antimicrobial administration.

Antimicrobial-resistant organisms found in humans are also found in companion animals, and there is increasing recognition of household sharing of resistant organisms.^1^

Dogs and their owners have frequent and intensive contact, which may increase the risk for transmission of zoonotic or antimicrobial-resistant organisms.^2^

Antimicrobials may exaggerate the risk of organism transmission due to the disruption of normal gastrointestinal flora and the enhanced potential for the development of antimicrobial resistance. Few published studies have specifically evaluated antimicrobial resistance in pet feces in relation to antimicrobial use, and of these none has assessed the association between antimicrobial-resistant flora in pets and their owners.^3–5^

We hypothesized that dogs receiving antimicrobials may develop antimicrobial-resistant bacterial species in their feces and that this may lead to shared resistant bacteria with the humans in their household. To establish proof of concept, we conducted a pilot study designed to isolate antimicrobial-resistant enteric bacteria from dogs and their owners before and after the dogs were treated with antimicrobials.

## Methods

The study protocol was approved by the Tufts University Institutional Review Board and by the Tufts Institutional Animal Care and Use Committee.

Pairs of dogs and their human owners were recruited at the Foster Hospital for Small Animals at Tufts University. Eligible dogs had been diagnosed with an infection warranting a 14-day course of amoxicillin-clavulanate. The paired human had to report frequent contact with their dog, with or without oral contact. The following exclusion criteria were applied regarding known or planned disruptions to gastrointestinal flora: (1) the dog experienced diarrhea in the prior week or was receiving oral antacids or probiotics; (2) the human used antimicrobials in the prior 60 days; (3) the human experienced diarrhea or abnormally loose stool in the prior week; or (4) any other humans or pets in the household planned to take antimicrobials during the dog’s antimicrobial course.

Stool samples were obtained from the dog and the human within 24 hours of antimicrobial initiation and again within 48 hours after antimicrobial completion. Owners were instructed to obtain specimens avoiding environmental contamination. Specimens were refrigerated at 4°C until transport to the laboratory and were frozen at −80°C within 24 hours of collection.

Stool samples were semiquantitatively cultured for enteric flora. Bacteria present at the time of antimicrobial initiation were deemed colonization flora, whereas new organisms at day 14 were termed acquisition flora. To screen for gram-negative resistance, MacConkey agar (MAC) with ampicillin-sulbactam (A/S), MAC with ciprofloxacin (CIP), and HardyChrom ESBL agar to detect extended-spectrum β-lactamase (ESBL) production were employed. For gram-positive screening, Mannitol Salt Agar with oxacillin was used to isolate methicillin-resistant *Staphylococcus aureus* (MRSA) and Bile Esculin Azide agar with vancomycin was used to detect vancomycin-resistant enterococci (VRE). Antimicrobial-free MAC and Tryptic Soy Agar blood agar plates were included as growth controls. Gram negative organisms were identified using API Strips [Biomeriux, Marcy-Etoile, France]. All isolate antimicrobial resistances were confirmed following Clinical and Laboratory Standards Institute guidelines using 3-5 colonies subcultured from the selective media.^6^ Pre-antimicrobial and post-antimicrobial samples were compared for changes in count and species of enteric flora.

Phenotypically similar organisms were analyzed by pulsed-field gel electrophoresis (PFGE) for genetic relatedness. Strains that displayed indistinguishable restriction fragment band patterns were defined as genetically identical.

## Results

Eight human–dog pairs were enrolled, each providing samples at day 1 and day 14 (Table 1). All samples had enteric flora present at the time of antimicrobial initiation, whereas 2 of the 8 dog samples had no detectable enteric flora on day 14. Of the 16 dog samples, 1 had insufficient quantity for full analysis; dog H on day 14 was unable to be tested for persistent *Escherichia coli* carriage.

**Table 1.**
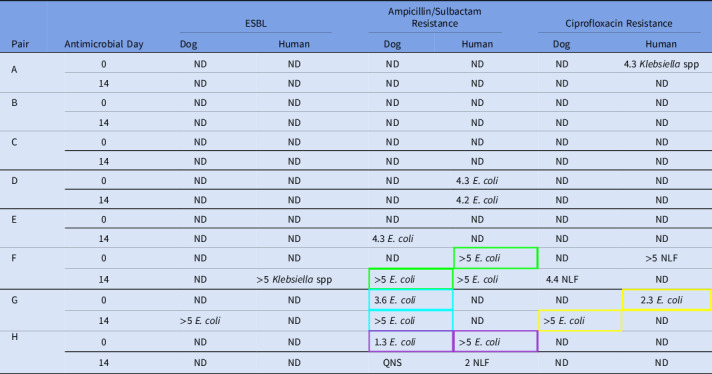
Baseline and acquired fecal colonization of antimicrobial-resistant organisms (log_10_ CFU/g stool)

Note. ND, not detected; ESBL, extended-spectrum β-lactamase producing organisms; NLF, non–lactose-fermenting organisms; QNS, quantity not sufficient. Isolates from pairs sharing a colored box outline displayed indistinguishable restriction fragment band patterns on pulsed-field gel electrophoresis and were considered genetically identical.

**Fig. 1.**
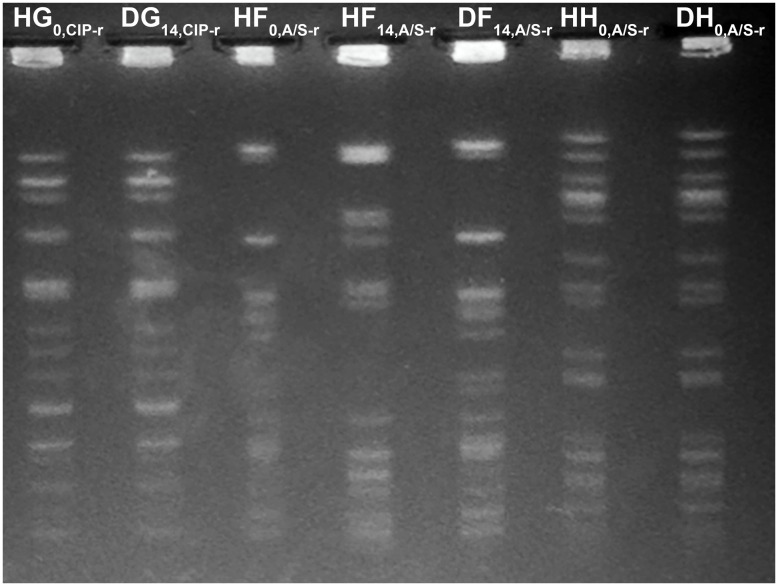
Pulsed-field gel electrophoresis for *E.coli* isolated from dog–human pairs. Isolates are identified by species (D, dog; H, human), pair (A–H), day (0 or 14), and plate type (CIP-r, ciprofloxacin resistant; A/S-r, ampicillin-sulbactam resistant). All matched pairs were identical (HG_0_ and DG_14_), (HF_0_ and DF_14_), and (HH_0_ and DH_0_). HF_14_ was unmatched to the other *E. coli* isolates from human and dog F.

No human or dog samples had detectible MRSA or VRE at any time point. The non–lactose-fermenting (NLF) bacteria detected in human F and dog F were unable to be grown in culture to determine species or assess for genetic relatedness. Pulsed-field gel electrophoresis results are shown in Figure 1. A genetically identical A/S-resistant *E. coli* was found in one human–dog pair at baseline (pair H). In two pairs, a resistant *E. coli* (A/S-resistant in pair F and CIP-resistant in pair G) identified in the owner at baseline was found in the dog at day 14, suggesting transmission from owner to pet (25% shared acquisition). One dog and one human from separate households acquired ESBL organisms, and 1 dog acquired A/S-resistant *E. coli* from a source other than their paired housemate.

Resistant-organism carriage changed in both owners and dogs over the study period: 5 (62.5%) of 8 humans and 2 (25%) of 8 dogs had antimicrobial-resistant flora at baseline compared to 3 (37.5%) of 8 humans and 3 (37.5%) of 8 dogs at day 14 after antimicrobial treatment. The only dog that had antimicrobial-resistant flora at day 0 that persisted to day 14, dog G, had a 2-log increase in organism count after the antibiotic course.

## Discussion

In this pilot study, we investigated shared carriage of enteric flora between dogs and their human owners when dogs are exposed to systemic antimicrobials. We detected evidence both of shared colonization at baseline (1 of 8 pairs, 12.5%) and of possible transmission of antimicrobial resistance from human to dog in 2 cases (25%). Additionally, we detected acquisition of new antimicrobial-resistant organisms from another source and increasing colony counts of antimicrobial-resistant organisms in dogs exposed to antimicrobials.

Many studies have suggested that pets may be able to act as a household reservoir for antimicrobial-resistant organisms.^3,4,7–10^ Interestingly in this study, the selective pressure of the oral amoxicillin-clavulanate may have facilitated transmission of antimicrobial-resistant organisms from humans to their pets. Further study is needed to determine the persistence of antimicrobial resistance in dogs at a later period after antimicrobials are discontinued.

Antimicrobial resistance as a shared trait within households can significantly affect the health of humans and companion animals, escalating the breadth of antimicrobial spectrum needed to treat both should they develop infection. Targeting antimicrobial stewardship efforts to companion animals and educating pet owners about the potential for transmission of antimicrobial-resistant bacteria is warranted.

This small study had several limitations, including the lack of a control group. It was more challenging to culture enteric flora when the dogs had recently recieved antimicrobials, resulting in no flora isolated in two dogs for their post-antimicrobial sample. Unabsorbed antimicrobial in the intestinal lumen may have sterilized cultures. Most samples were collected by the subjects; therefore, there may have been some contamination or mishandling of samples. We were also limited by the quantity of certain samples. Given the small study population, each sample weighed heavily into the overall results. As a pilot study, we only implemented standard culture-based organism detection. More sensitive molecular methods, such as whole genome sequencing or pyrosequencing, may better characterize the complete microbiome and resistome and comare genetic relatedness.

In this study, we identified changes in antimicrobial-resistant organisms in pet dogs and their owners after the dogs received antimicrobials. Treating dogs with antimicrobials may affect the resistome of the household. Additional studies are needed to further elucidate the role of antimicrobial administration in animals on the development and sharing of antimicrobial-resistant bacteria within households, as well as the effect of antimicrobial type and treatment duration.
